# The Development of Context-Sensitive Attention in Urban and Rural Brazil

**DOI:** 10.3389/fpsyg.2020.01623

**Published:** 2020-07-24

**Authors:** Pablo Mavridis, Joscha Kärtner, Lília Iêda Chaves Cavalcante, Briseida Resende, Nils Schuhmacher, Moritz Köster

**Affiliations:** ^1^Department of Psychology, University of Münster, Münster, Germany; ^2^Department of Behavior Theory and Research, Federal University of Pará, Belém, Brazil; ^3^Department of Experimental Psychology, University of São Paulo, São Paulo, Brazil; ^4^Department of Cross-Cultural Developmental Psychology, Free University Berlin, Berlin, Germany

**Keywords:** holistic and analytic perception, context-sensitivity, development of visual attention, cognitive development, cross-cultural comparison, urban versus rural context

## Abstract

Human perception differs profoundly between individuals from different cultures. In the present study, we investigated the development of context-sensitive attention (the relative focus on context elements of a visual scene) in a large sample (*N* = 297) of 5- to 15-year-olds and young adults from rural and urban Brazil, namely from agricultural villages in the Amazon region and the city of São Paulo. We applied several visual tasks which assess context-sensitive attention, including an optical illusion, a picture description, a picture recognition and a facial emotion judgment task. The results revealed that children and adults from the urban sample had a higher level of context-sensitive attention, when compared to children and adults from the rural sample. In particular, participants from São Paulo were more easily deceived by the context elements in an optical illusion task and remembered more context elements in a recognition task than participants from rural Amazon villages. In these two tasks, context-sensitivity increased with age. However, we did not find a cultural difference in the picture description and the facial emotion judgment task. These findings support the idea that visual information processing is highly dependent on the culture-specific learning environments from very early in development. Specifically, they are more consistent with accounts that emphasize the role of the visual environment, than with the social orientation account. However, they also highlight that further research is needed to disentangle the diverse factors that may influence the early development of visual attention, which underlie culture-specific developmental pathways.

## Introduction

Over the past decades it has been established that human perception differs markedly between cultures. In their seminal work, Masuda and Nisbett (2001) investigated how North American and East Asian participants differ in their perception of visual scenes and have described two prototypical perception styles, North Americans being more analytic, primarily focusing on focal objects of a scene, and East Asians being more holistic, showing a higher sensitivity for context elements of a visual scene (also defined as low versus high context-sensitivity).

These differences in visual perception have been documented in a number of studies and across several experimental paradigms. In particular, Masuda and Nisbett (2001) found that US-Americans tended to report a focal fish swimming in an aquarium, while Japanese participants described and remembered more details from the background, including plants and smaller animals in an aquarium scene. Furthermore, Doherty et al. (2008) found that Japanese participants are more easily deceived by context elements in optical Ebbinghaus illusions, namely when adjusting a focal element within a deceptive context, in contrast to participants from the United Kingdom. Regarding the perception of social stimuli, Masuda et al. (2008) found that Japanese participants were influenced by context information to a higher degree than North Americans: In a facial emotion judgment task participants were asked to judge the emotion of a person presented in the center of a social scene, surrounded by people with the same or a different emotion. Participants from Japan adjusted their judgment of the emotion of the focal person more strongly to the emotions of the people in the background, when compared to participants from the United States. These studies have shown consistent cross-cultural differences in visual perception, with a higher context-sensitive attention in participants from Eastern compared to Western cultural groups.

This raises the critical question, when and how cultural differences in visual perception develop. It has been suggested that the social construction of attention plays a central role in the ontogeny of different perception styles (Masuda and Nisbett, 2001; Nisbett and Masuda, 2003), such as the way close others guide the attention early in development (Senzaki et al., 2016; Köster and Kärtner, 2018). Focusing on cultural models more generally, the social orientation hypothesis (Varnum et al., 2010) posits that holistic perception is associated with an interdependent cultural model, with an emphasis on the individual as a part of their social group. In contrast, an analytic perception style is associated with an independent cultural model, with an emphasis on an individual’s autonomy (Markus and Kitayama, 1991). This is consistent with findings from classical comparisons of visual perception between the United States (being a prototypical independent culture) and East Asian cultural groups (being a prototypical interdependent culture), as reported above. However, another theoretical account, the visual environment hypothesis, emphasizes that the visual and physical environment differs between North American and East Asian cities (Miyamoto et al., 2006; Morling and Lamoreaux, 2008). For example, urban environments in East Asian cities are visually more complex and therefore afford higher levels of context-sensitivity (Miyamoto et al., 2006). These differences in the physical environment may likewise influence the development of visual perception between those cultural groups.

Concerning its early development, Imada et al. (2013) describe that context-sensitive attention develops in the early school years. In their study, 6- to 7-year-olds from Kyoto, Japan, showed significantly higher levels of context-sensitive attention than children of the same age from Minneapolis, United States, in an overall score, which was the aggregated result of a picture description task and an Ebbinghaus illusion. This difference between the two cultural groups increased with age, throughout the early school years. However, in the picture description task, cross-cultural differences were already present in 4- to 5-year-olds when analyzing children’s references to context elements versus focal elements. Studies testing other aspects of visual cognition provided evidence that cultural differences may emerge even earlier (Kuwabara and Smith, 2012, 2016).

In recent developmental studies on cross-cultural differences in context-sensitive attention, a specific focus lied on the comparison between participants from rural and urban environments (Bremner et al., 2016; Jurkat et al., 2020; Köster et al., 2018). Bremner et al. (2016) reported that children from a traditional Himba society in rural Namibia showed significantly less deception in an Ebbinghaus illusion task than did children growing up in the nearest urban settlement in Namibia or children growing up in urban United Kingdom. In another study, Köster et al. (2018) applied an Ebbinghaus illusion task with 5-year-olds from three different cultural groups (urban Germany, rural Cameroon, urban Japan) and found significantly less deception in an optical illusion task for children from rural Cameroon than for the two urban samples. The same study also found a higher object focus in the rural sample from Cameroon in an eye-tracking paradigm. However, there were no differences between children from an independent culture (Germany) versus children from an interdependent culture (Japan) in these tasks, at this age. These studies provide first evidence that context-sensitive attention, as measured with classical paradigms, may be higher in children from urban compared to rural samples, and closely resemble the results of much earlier studies conducted with adult participants (Segall et al., 1966; Jahoda and Stacey, 1970).

Studies that compare urban versus rural environments are a critical extension to the contrast between participants from Eastern and Western samples. Specifically, the differences reported in the studies above can less well be interpreted within the social orientation explanatory framework, because individuals from rural environments are commonly described as being socially more closely oriented toward each other than in urban environments (Greenfield et al., 2003; Kagitcibasi, 2005; Keller, 2007). They are, on the other hand, compatible with a visual environment hypothesis, because it is likely that urban contexts are more complex than rural environments in terms of physical and social affordances (Miyamoto et al., 2006; Morling and Lamoreaux, 2008; Linnell and Caparos, 2019). Note that theoretical accounts on cultural variation in human development have long emphasized the role of differences in the physical and social affordances between urban and rural settings (Greenfield et al., 2003; Keller, 2007). However, to date, this line of research has mainly focused on rural settings on the African continent, in contrast to urban samples in the United States and European countries. It is thus a critical question, and may further inform the different accounts on the development of visual perception styles, if the differences between urban versus rural cultural environments would generalize to participants from further urban and rural environments.

Toward this end, in the present study, we provide data for an urban and a rural environment in Brazil, two populations which, to our knowledge, have not yet been investigated in terms of the development of visual perception styles. Specifically, we compare the development of context-sensitive attention between urban São Paulo and rural subsistence-based villages in the Brazilian Amazon region near Belém, in a variety of experimental tasks. We know from former studies that these urban and rural environments within Brazil differ not only in terms of their social and ecological (i.e., environmental) structure, but also in their cultural orientation (Seidl-de-Moura et al., 2008; Köster et al., 2016). Namely, participants from urban Brazil typically emphasize an autonomous development (i.e., independence), while participants in rural Brazil emphasize a relational cultural model (i.e., being conceptually closer to an interdependent cultural model). In order to characterize differences in context-sensitivity and their ontogenetic development, we collected the data from a large number of children between 5 and 15 years of age and, in addition, of adults between 20 and 30 years of age, to estimate the developmental end points. We applied a set of tasks, which had already been successfully used in studies investigating the development of visual perception styles (Masuda et al., 2008; Köster et al., 2017, 2018). We used a picture description task, an Ebbinghaus illusion task, a picture recognition task, and a task focusing on the judgment of facial emotions. We chose those four paradigms, because all of them have formerly been applied in studies comparing context-sensitive perception between cultural groups. While the social orientation hypothesis would predict higher context-sensitivity in the rural villages in the Amazon region, according to the visual environment hypothesis, one may expect higher degrees of context-sensitivity for the sample from São Paulo. Furthermore, we hypothesized that cross-cultural differences would develop throughout the school years in the sample of children, and to be specifically pronounced in the adult sample.

## Materials and Methods

### Participants

The final sample consisted of 297 participants. We assessed 131 children between 5 and 15 years of age (*M* = 10;10, *SD* = 3;18, 49% females) and 32 adults between 20 and 30 years of age (*M* = 24;25, *SD* = 3;64, 69% females) in rural agricultural villages in the municipality of Castanhal in the state of Pará in northern Brazil. Three additional adults were excluded from analysis because they grew up in nearby urban settlements. Furthermore, we assessed 103 children (*M* = 9;42, *SD* = 2;87, 54% females) and 31 adults (*M* = 23;52, *SD* = 3;08, 52% females) from capital São Paulo (metropolis in southern Brazil). Two additional children in São Paulo participated but were excluded from analysis because their parents reported that they were from a rural region in São Paulo state (*n* = 1) or adopted (*n* = 1). One adult did not specify their age. It was estimated by substituting with the sample mean. Not all participants completed the full set of tasks. Thus, analyses for each task are based on the subset of participants that completed a specific task. For all analyses, the samples were split into four age groups, 5- to 7-year-olds, 8- to 11-year-olds, 12- to 15-year-olds, with the fourth age group comprised of adults between 20 and 30 years of age. This strategy allows for more specific conclusions and interpretations of differences between relevant age groups (see Imada et al., 2013, for a similar approach), because we did not necessarily expect linear developmental trajectories of context-sensitivity. In the rural sample, the number of children in each age group was 34 in the group of 5- to 7-year-olds, 48 in the group of 8- to 11-year-olds and 49 in the group of 12- to 15-year-olds. In the urban sample the distribution was 28 in the group of 5- to 7-year-olds, 47 in the group of 8- to 11-year-olds and 28 in the group of 12- to 15-year-olds.

The recruitment in rural Brazil was done in cooperation with local healthcare centers and testing was conducted in schools or in families’ homes. In São Paulo children were recruited and assessed in *Parque Villalobos*, a spacious urban park located in a neighborhood primarily inhabited by middle class and upper-middle class families. However, the park is also commonly visited by families from different parts of the city and its outskirts, making the sample more heterogenous in terms of socio-economic status. Adults from São Paulo were recruited and participated on the University of São Paulo campus and were mostly students.

Each adult participant and each parent of the underaged participants gave their informed written consent before participating in the study. Furthermore, we obtained informed assent from each child prior to testing.

### Stimuli and Procedure

The experiment included four different visual attention tasks, an optical illusion task, a picture description task, a picture recognition task, and a task involving judgment of facial emotions. Tasks were presented in this order (fixed) to avoid different carry-over effects between participants.

For the rural sample, all testing was done by the first author, fluent in Portuguese. In São Paulo about half of the child sample was assessed by the first author while the other half, as well as the adult sample was conducted by a local research assistant. Stimuli presentation and data recording were conducted on laptop computers, using a customized offline version of Labvanced (Finger et al., 2017). Stimuli were presented on a 15.6° screen or a 14° screen, but with the same absolute presentation size of stimuli on both screens. The distance between participants and the monitor was kept constant, at around 50 cm.

#### Optical Illusion Task

**FIGURE 1:**
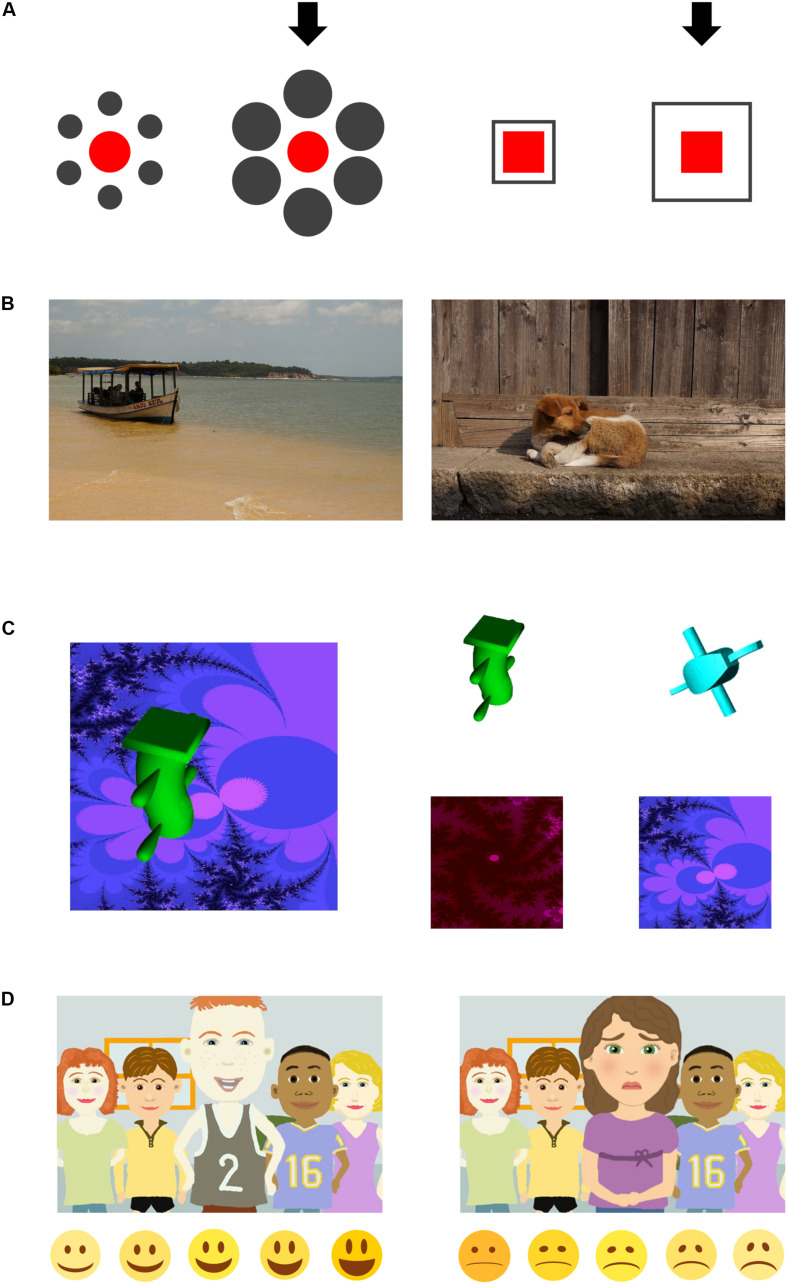
Example stimuli for the applied tasks. **(A)** Optical illusion task. The arrows in the two different types of illusion trials indicate the side on which the red target element was adjusted by the participants. **(B)** Picture description. Subjects were asked to verbally describe images depicting either animals or means of transport. **(C)** Recognition task. The left panel shows an exemplary stimulus presented during the first phase. The panels on the right show the two-alternative-forced-choice options for object (top) and background (bottom) presented during the second phase. **(D)** Judgment of facial emotions. Participants saw stimuli with happy (left) or sad (right) focal persons. After each stimulus, a corresponding emoticon-scale was presented (bottom).

Participants were presented with two variants of the Ebbinghaus illusion (Köster et al., 2018 for the use of a similar set of tasks). The participant was asked to adjust the size of a red-colored target shape, which was indicated by a black arrow, until it matched the size of a reference shape which was also colored in red. Both shapes were surrounded by deceiving context elements colored in gray (see Figure 1A). Shapes were adjusted via two keys on a keyboard. Both versions of the illusion were presented in two variations, with the target element being surrounded either by the smaller context elements or the larger context elements. These variations were then displayed with the target element on either side of the screen. Thus, the resulting number of trials was 4 for each version and a total of eight trials, presented in a fixed order.

Participants were instructed to not attend to the context, but only to the red elements, to avoid a relative interpretation of the task instruction. Before being presented with the actual illusion trials, participants completed four warm-up trials introducing the task with the same instruction (to adjust the size of the target element until it matches the reference element), but without the presence of gray context elements. These warm-up trials were also used as a measure for the accuracy of the participants’ size adjustments.

We summarized the results into a single context-sensitivity score for each participant, namely the mean deception over all trials of each version of the Ebbinghaus illusion. Specifically, the mean deception was computed by subtracting the mean deviation between target and reference element in illusion trials with context elements (in percent) from the mean deviation in trials without context elements (in percent). In other words, the context-sensitivity score reflects the difference between the adjusted element and the red reference element in percent, corrected for the participants’ accuracy. Higher scores on this measure indicate a higher context-sensitivity.

Participants, whose mean degrees of deception in trials without context stimuli were higher than 10% or deviated by more than 3*SD* from the mean of the participants’ respective age group, were excluded from analysis due to insufficient accuracy. We excluded *n* = 3 children from the youngest age group in São Paulo and *n* = 1 adult from São Paulo.

#### Picture Description Task

Participants were shown eight photographs containing a focal object (animals and means of transport) in front of a background (e.g., natural scenes or buildings), see Figure 1B. Pictures were selected in consultation with local researchers and based on our experiences with previous adoptions of this paradigm, ensuring that participants from both cultural groups would be familiar with the presented stimuli. Participants were instructed to describe what they saw on the pictures (“Please describe to me what’s in the pictures”) to the experimenter, who was sitting on the other side of the screen, allegedly not knowing the pictures himself. Each picture was presented for 15 s.

The descriptions were audio recorded and later coded for the number of references to the focal object and its features (e.g., boat, has people on it) and the background and its features (e.g., the sky, it’s blue), using (MAXQDA, 2019). In order to quantify participants’ context-sensitivity in verbal descriptions, we computed a context-sensitivity score: All references to the background and its features were divided by the sum of all references to the object and the background, including features. Thus, a score of 1 would indicate that a participant only talked about the background, while a score of 0 would indicate that a participant only referred to the object. Inter-rater agreements for 18% of the data were sufficiently high (rural sample: κ_object_ = 0.81, κ_background_ = 0.76; urban sample: κ_object_ = 0.82, κ_background_ = 0.81). Cohen’s kappas were calculated using the Brennan and Prediger method (Brennan and Prediger, 1981). The timestamps for corresponding codes had to overlap at least 33% to be considered congruent between coders.

Participants who misunderstood the task or whose context-sensitivity score deviated by more than 3*SD* from the mean of the participants’ respective age group, were excluded from analysis. For the rural sample we excluded *n* = 1 children from the youngest age group, *n* = 2 children from the 8- to 11-year-old age group, *n* = 3 children from the 12- to 15-year-old age group, and *n* = 1 adult. For the urban sample we excluded *n* = 2 children from the 8- to 11-year-old age group.

#### Picture Recognition Task

Participants were shown a set of 16 abstract images, each presented for 3 s. Stimuli consisted of fractal pictures (created with Quadrium, 2009) as backgrounds, and abstract, non-semantic objects retrieved from an online database as salient focal objects (see Figure 1C). Similar stimuli have been adopted in a previous study by Köster et al. (2018). We chose abstract, non-semantic stimuli in order to avoid possible confounding effects caused by semantic stimuli. Subjects were instructed to look at the pictures attentively because the experimenter would later “ask a few questions about these pictures.” Stimuli were presented in a fixed order.

In a subsequent retrieval phase, participants were shown stimuli pairs of either focal objects or backgrounds. Each pair consisted of a previously presented focal object/background and either an entirely new object/background or a modified version of the same object/background serving as a distractor. For each pair, the participants were instructed to select the object or background they had seen during the presentation phase, in a two-alternative-forced-choice paradigm. The stimuli pairs were presented in a fixed order, according to the prior presentation phase. Stimuli were modified using (GNU, 2012).

The context-sensitivity measure for the picture recognition task was computed by dividing the number of correctly recognized background stimuli by the total number of correctly recognized stimuli (objects + backgrounds), with a higher score indicating higher context-sensitivity. As a control measure, we analyzed overall recognition performance of the subjects and determined a higher than chance (>50%) rate recognition of target stimuli as the threshold for sufficient memory performance which would qualify the participant’s data to be further analyzed. Prior to analysis we excluded seven children (rural sample: *n* = 3 from the youngest age group and *n* = 1 out of the 8- to 11-year-olds; urban sample: *n* = 3 from the youngest age group) because they failed to recognize the presented stimuli above chance (>50%).

#### Judgment of Facial Emotions

In the facial emotion judgment task, participants saw 24 social stimuli containing a group of five cartoon-style people expressing sadness or happiness (see Figure 1D). One salient person stood in the foreground while four others stood in the background. Each stimulus was presented for 5 s and then followed by a screen showing five emoticons depicting either sadness or happiness (depending on the emotion of the central person on the previous picture), at five different levels of intensity (see Figure 1D). Participants were instructed to “tell (the experimenter) how the person in the middle is feeling” by pointing at or clicking on the emoticon which best described the figure’s emotion.

Emotions were either congruent or incongruent between the focal person and the background persons. This is, they either showed the same emotion or the oppositional emotion. The social stimuli came from a study by Masuda et al. (2008). For the purpose of this study we selected the stimuli which matched Brazilian ethnicities best, not using the entire stimuli pool. The emoticon-scale was created using Microsoft PowerPoint. We developed it with the youngest participants in mind and with having in mind that in rural Brazil responding to finely graded numerical scales is very uncommon also for older children and adolescents, based on a former study the research group conducted in the same region (Köster et al., 2016).

Following the hypothesis and results of Masuda et al. (2008), congruence between the background figures’ emotion and the central figure’s emotion should lead to this emotion being perceived more intensely and thus receiving ratings of higher intensity, more so in individuals with higher context-sensitive attention. Context-sensitivity measures for this task were computed by calculating sum scores for each of the four different levels: (a) happy and congruent background, (b) happy and incongruent background, (c) sad and congruent background, (d) sad and incongruent background. We calculated two distinct measures of context-sensitivity for the happy and the sad focal faces by subtracting the incongruent sum scores from the congruent sum scores. Higher measures index higher context-sensitivity.

Prior to the analysis we excluded *n* = 12 subjects because they either had obvious difficulties understanding the task or almost exclusively chose the highest degree of intensity on the emoticon scales in their answers. Ten of the excluded subjects were children from the rural sample (*n* = 5 out of the youngest age group, *n* = 4 out of the 8- to 11-year-old age group and *n* = 1 out of the 12- to 15-year-old age group) and *n* = 2 were children from São Paulo (*n* = 1 out of the youngest age group and *n* = 1 out of the 8- to 11-year-old age group). Explorative analyses of the four sum scores revealed no extreme outliers deviating >3*SD* from the mean of the participants’ respective age group, such that there were no further exclusions.

## Results

### Optical Illusion Task

**FIGURE 2:**
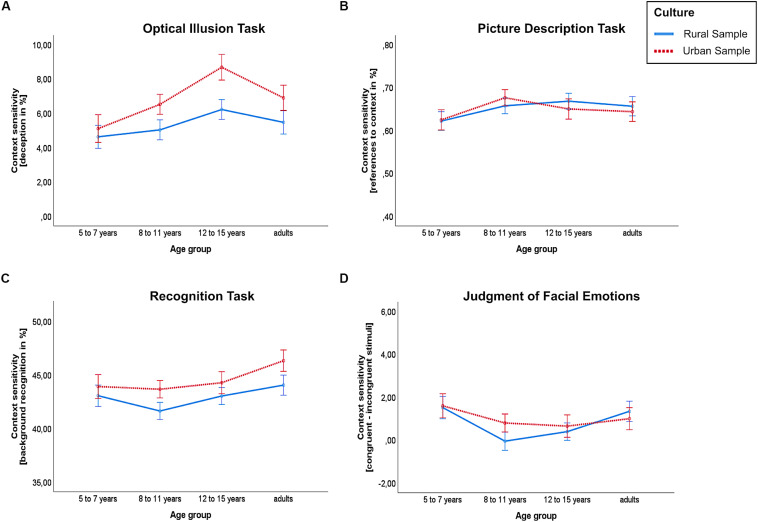
Context-sensitivity scores by culture and age group for **(A)** the Ebbinghaus illusion, **(B)** the picture description, **(C)** the recognition task and **(D)** the judgment of facial emotions (happy stimuli set). Higher values indicate higher context-sensitivity. Whiskers depict one standard error of the mean.

For the participants’ deception in the illusion task, we conducted a 4 (age group) × 2 (cultural group) ANOVA. This revealed significant main effects for age group, *F*(3,285) = 4.816, *p* = 0.003, η^2^ = 0.048, and cultural group, *F*(1,285) = 8.185, *p* = 0.005, η^2^ = 0.028, but no interaction between age group and cultural group, *F*(3,285) = 0.705, *p* = 0.550, see Figure 2A. Across cultures, the mean deception was lowest in the group of 5- to 7-year-olds (*M* = 4.9%) and increased with age throughout the children and adolescent sample: 8- to 11-year-olds (*M* = 5.7%), 12- to 15-year-olds (*M* = 7.5%). However, the mean was descriptively lower in the adult group than in the group of 12- to 15-year-olds (*M* = 6.3%). Bonferroni corrected pairwise comparisons revealed significant differences between 5- to 7-year-olds and 12- to 15-year-olds (−2.602, 95%-CI[−4.511, −0.694], *p* = 0.002) and between 8- to 11-year-olds and 12- to 15-year-olds (−1.759, 95%-CI[−3.447, −0.071], *p* = 0.036).

### Picture Description Task

A 4 (age group) × 2 (cultural group) ANOVA revealed no significant main effect for age group, *F*(3,277) = 1.602, *p* = 0.189, or cultural group, *F*(1,277) = 0.029, *p* = 0.864. Furthermore, the interaction effect between age group and cultural group was non-significant, *F*(3,277) = 0.435, *p* = 0.728, see Figure 2B.

When comparing the volume (the total number of references), we found a main effect for age group, *F*(3,277) = 24.202, *p* < 0.001, η^2^ = 0.208, and cultural group, *F*(1,277) = 34.948, *p* < 0.001, η^2^ = 0.112, and an interaction between age group and cultural group, *F*(3,277) = 4.704, *p* = 0.003, η^2^ = 0.048. Specifically, the volume increased with age and this increase was higher in the urban sample compared to the rural villages. The mean volumes in the rural sample were as follows: *M* = 31.3 in the group of 5- to 7-year-olds, *M* = 33 in the group of 8- to 11-year-olds, *M* = 36 in the group of 12- to 15-year-olds and *M* = 42 in the adult group. In contrast, the mean volumes in the urban sample were *M* = 32.7 in the group of 5- to 7-year-olds, *M* = 40.7 in the group of 8- to 11-year-olds, *M* = 46 in the group of 12- to 15-year-olds and *M* = 61.3 in the adult group.

### Recognition Task

For participants’ recognition of background versus object elements, we found a significant main effect for age group, *F*(3,279) = 3.175, *p* = 0.025, η^2^ = 0.033, and cultural group, *F*(1,279) = 4.438, *p* = 0.036, η^2^ = 0.016, but no interaction between age group and cultural group, *F*(3,279) = 0.213, *p* = 0.888, see Figure 2C. Across cultures, the mean context-sensitivity score was descriptively lower in the group of 8- to 11-year-olds (*M* = 42.6%) than in the youngest age group (*M* = 43.6%) and in the group of 12- to 15-year-olds (*M* = 43.5%), while it was descriptively highest in the adults group (*M* = 45.4%), see Figure 2C. A Bonferroni corrected pairwise comparison revealed a significant difference between 8- to 11-year-olds and adults (−2.792, 95%-CI [−5.197, −0.388], *p* = 0.013).

### Judgment of Facial Emotions

The context-sensitivity measures for the sad stimuli set and for the happy stimuli set did not correlate significantly (*r* = −0.015, *n* = 279, *p* = 0.807). Hence, we analyzed them separately. Exemplarily, we plotted the results for the happy stimuli set (see Figure 2D). A corresponding graph, depicting the ANOVA results for the sad stimuli set, is provided in the Supplementary Material.

For the sad stimuli, we found no significant main effect for age group, *F*(3,271) = 1.028, *p* = 0.38, or cultural group, *F*(1,271) = 0.164, *p* = 0.686. The interaction between age group and cultural group was also non-significant, *F*(3,271) = 0.094, *p* = 0.963.

However, for the happy stimuli, the ANOVA revealed a significant main effect for age group, *F*(3,271) = 2.852, *p* = 0.038, η^2^ = 0.031. The main effect for cultural group was non-significant, *F*(1,271) = 0.191, *p* = 0.662, as was the interaction between age group and cultural group, *F*(3,271) = 0.529, *p* = 0.663, see Figure 2D.

Across cultures, the mean context-sensitivity score for the happy stimuli set was descriptively lower in the group of 8- to 11-year-olds (*M* = 0.33) and in the group of 12- to 15-year-olds (*M* = 0.46) than in the youngest age group (*M* = 1.56) and in the group of adults (*M* = 1.21). Bonferroni corrected pairwise comparisons revealed no significant differences between age groups.

### Correlations Between Task Measures

We calculated partial correlations between the task measures for each cultural group, controlling for participants’ age. All correlations were based on the maximum number of observations available for both measures. Only 2 out of 20 correlations reached *p* < 0.05 (uncorrected), see Table 1. The reported correlations are uncorrected and serve illustration purposes.

**TABLE 1:** Correlations between task measures split by sample. Measures for the two scales in the judgment of facial emotions task are reported separately.

**Context sensitivity score**	**1.**	**2.**	**3.**	**4.**
**Rural sample**				
1. Optical illusion				
2. Picture description	0.02			
3. Picture recognition	0.02	0.11		
4. Emotion judgment (happy scale)	0.08	–0.05	0.15	
5. Emotion judgment (sad scale)	0.06	0.06	–0.08	−0.01
**Urban sample**				
1. Optical illusion				
2. Picture description	–0.09			
3. Picture recognition	–0.05	0.08		
4. Emotion judgment (happy scale)	−0.18*	0.06	–0.12	
5. Emotion judgment (sad scale)	0.12	0.17	0.19*	−0.02

The table displays Pearson’s correlation coefficients, corrected for age (partial *r*). *p < 0.05, uncorrected.

### Comparisons Between Cultures Split by Age Group

Note that the non-significant interactions did not call for subsidiary *t*-tests, split by age groups. However, these tests are provided for each task in the Supplementary Material.

## Discussion

Overall, the results of the present study revealed that children and adults from urban São Paulo showed a higher level of context-sensitivity compared to participants from rural villages in the Brazilian Amazon region. Namely, we found that children and adults from São Paulo were more easily deceived by context elements in the optical illusion task and remembered more context elements in the recognition task, when compared to children and adults from the rural villages. Across both tasks, context-sensitivity increased with age. However, we did not find a cultural difference in the picture description and the facial emotion judgment tasks. There were no consistent correlations between the different tasks, which is consonant with findings on similar tasks in studies contrasting adult participants from East Asia and the United States (Na et al., 2010) and children from rural and urban environments (Köster et al., 2018).

The results of the optical illusion and recognition tasks resemble former developmental studies that compared differences in perception styles between urban and rural environments (Bremner et al., 2016; Köster et al., 2018). While this former research focused on samples from rural environments on the African continent and urban samples in the United States or Europe, the present study extends these findings to the South American continent. Thus, the present findings further support theoretical frameworks that emphasize the relevance of the visual environment for cultural differences in perception styles (Miyamoto et al., 2006; Morling and Lamoreaux, 2008). Like former studies, these findings do not suggest that the social orientation hypothesis (Varnum et al., 2010) generalizes to cultural contrast beyond the comparison between East Asian and United States populations (cf. Jurkat et al., 2020).

Several ideas have been put forward to explain these differences in context-sensitivity between rural and urban environments. An initial proposal mainly focused on cultural differences in optical illusions in adult participants, emphasizing that individuals in urban environments are exposed to more “carpentered-corners”, which make spatial perception more reliant on geometrical shapes, and may thus lead to a higher degree of deception in optical illusion tasks (Segall et al., 1966; Jahoda and Stacey, 1970; Henrich et al., 2010). However, it is not clear how this explanation accounts for memory effects of elements of visual scenes in the present study or cultural differences in picture descriptions found in a former study (Köster et al., 2018). Therefore it is critical to look at further differences between urban and rural environments that could possibly explain cross-cultural differences in the development of perception styles (Miyamoto et al., 2006; Morling and Lamoreaux, 2008). From a general viewpoint, visually and physically more complex environments may afford higher levels of context-sensitivity (Miyamoto et al., 2006). In a more specific account, it has been argued that diverse factors in urban environments lead to a more explorative information processing and higher levels of context-sensitivity, opposed to a more task-focused perception style in rural samples (Linnell et al., 2013; Linnell and Caparos, 2019). This line of research suggests that this effect is mediated by stress factors related to urban living (Lederbogen et al., 2011) leading to an increased neurophysiological activation linked to arousal processes and attentional states (Linnell and Caparos, 2019).

Another, more methodologically oriented, explanatory account is that participants from urban environments are generally more familiar with the visual stimuli in the commonly applied tasks (Jurkat et al., 2020; Köster et al., 2018) which allows them to process stimuli more efficiently, first the object, and then the context information. In accordance with this proposal, it has recently been shown that context-sensitive attention measures depend on the type of stimuli used, and that results may look different, tilted more toward the social orientation hypothesis, when stimuli are more carefully adjusted to the specific environment they are tested in Jurkat et al. (2020). Possibly, this may explain why we did not find a difference in context-sensitive attention in the picture description task in the present study, where we explicitly used pictures that were familiar to participants from both urban and rural Brazil.

Other than expected, in the optical illusion and the recognition task, we did not find a differential development between the two cultural groups. That is, although the cultural difference is descriptively less pronounced in the youngest age group (5- to 7-year-olds), the analyses did not reveal significant interaction effects between culture and age. This is in line with former studies contrasting rural and urban populations (Bremner et al., 2016; Köster et al., 2018) in the sense that cultural differences in perception between urban and rural environments were present earlier, compared to studies that investigated cultural differences in perception along the independent versus interdependent dimension (Imada et al., 2013). Thus, rural versus urban differences in visual perceptual development may follow a different developmental trajectory than the East-West contrast and, in consequence, may potentially rely on different developmental processes. Furthermore, we neither found a cross-cultural difference in context-sensitivity in the picture description, nor in the facial emotion judgment task. Noteworthy, in the picture description task, participants from São Paulo were verbally more fluent – i.e., the total number of references was larger in this sample – and this difference emerged with age, two effects which both may have affected the present results. For the facial emotion judgment task, it remains unclear whether it captures the same aspects of context-sensitivity as measured in the more perceptually based tasks. Alternatively, the differences found between Westerners and East Asians may indeed rely on more socially constructed differences in cognition, associated with independence and interdependence (Masuda et al., 2008).

Overall, we found a differential pattern of results between tasks and also no consistent evidence for a correlation between tasks, which corresponds to the findings of former studies (Na et al., 2010; Köster et al., 2018). Thus, cross-cultural differences in visual attention are highly task- and stimulus-dependent (see also Jurkat et al., 2020; Köster et al., 2018) and their early development seems to be more complex than previously assumed. In particular, both environmental affordances and social orientation may contribute, each in their own ways, to different aspects of children’s perceptual development. It may thus be critical for future research in developmental science, to take into account different attention systems (Posner and Petersen, 1990; Raz and Buhle, 2006) and to identify different developmental processes that shape different facets of human visual attention across cultures (Köster and Kärtner, 2019). For instance, it is important to better understand early visual experiences children are exposed to in different cultures (Miyamoto et al., 2006; see also Kuwabara et al., 2020), but also specific social interaction experiences, which may lead to different developmental pathways (Keller and Kärtner, 2013). For example, the way close others structure children’s visual attention in early development and thereby adjust their attentional focus to different elements of the physical and social environment (Köster and Kärtner, 2018; Rogoff, 2003). Further research is certainly needed to disentangle the diverse factors that shape cross-cultural and inter-individual differences in human cognition throughout development.

## Data Availability Statement

All datasets generated for this study are included in the Supplementary Material.

## Ethics Statement

Ethical review and approval was not required for the study on human participants in accordance with the local legislation and institutional requirements. Written informed consent to participate in this study was provided by the participants’ legal guardian/next of kin.

## Author Contributions

PM, JK, and MK conceptualized the study. PM, NS, and MK designed the study. PM conducted the study with the support of LC and BR. PM and MK analyzed the data and wrote the manuscript. All authors provided critical feedback to the manuscript. MK supervised the study.

## Conflict of Interest

The authors declare that the research was conducted in the absence of any commercial or financial relationships that could be construed as a potential conflict of interest.
