# Supplementary material for: The Development of Context-Sensitive Attention in Urban and Rural Brazil
Source: Front Psychol. 2020 Jul 24;11:1623. doi: 10.3389/fpsyg.2020.01623 (PMC7393234; doi:10.3389/fpsyg.2020.01623)
Supplement: Supplementary file 2 [file Table_1.docx]

Supplementary Material

# Supplementary Data

Since we did not find any significant interaction when running the ANOVAs, we did not report comparisons between cultural groups split by age group in the main text. Find below the *t*-test results for comparisons between the cultural groups in each age group for each task. The only difference significant to *p* < 0.05 is marked with an asterisk: in the Ebbinghaus illusion, 12- to 15-year-olds from São Paulo were significantly more deceived by context elements than adolescents from the same age group in the rural sample.

Ebbinghaus illusion task:
5- to 7-year-olds: *t*(59) = -0.504, *p* = 0.616
8- to 11-year-olds: *t*(93) = -1.980, *p* = 0.051
12- to 15-year-olds: t(75) = -2.055, *p* = 0.043*
adults: *t*(61) = -.1.380, *p* = 0.173

Picture description task:
5- to 7-year-olds: *t*(60) = -0.111, *p* = 0.912
8- to 11-year-olds: *t*(89) = -0.400, *p* = 0.690
12- to 15-year-olds: *t*(72) = -0.695, *p* = 0.490
adults: *t*(61) = -0.498, *p* = 0.620

Recognition task:
5- to 7-year-olds: *t*(54) = -0.624, *p* = 0.535
8- to 11-year-olds: *t*(92) = -1.720, *p* = 0.089
12- to 15-year-olds: *t*(76) = -0.991, *p* = 0.325
adults: *t*(64) = -1.587, *p* = 0.117

Judgment of facial emotion task (sad stimuli):
5- to 7-year-olds: *t*(52) = -0.382, *p* = 0.704
8- to 11-year-olds: *t*(86) = -0.346, *p* = 0.730
12- to 15-year-olds: *t*(75) = -0.012, *p* = 0.990
adults: *t*(63) = -0.410, *p* = 0.683

Judgment of facial emotion task (happy stimuli):
5- to 7-year-olds: *t*(52) = -0.089, *p* = 0.929
8- to 11-year-olds: *t*(86) = -1.283, *p* = 0.203
12- to 15-year-olds: *t*(75) = -0.470, *p* = 0.639
adults: *t*(63) = 0.592, *p* = 0.556

# Supplementary Figures


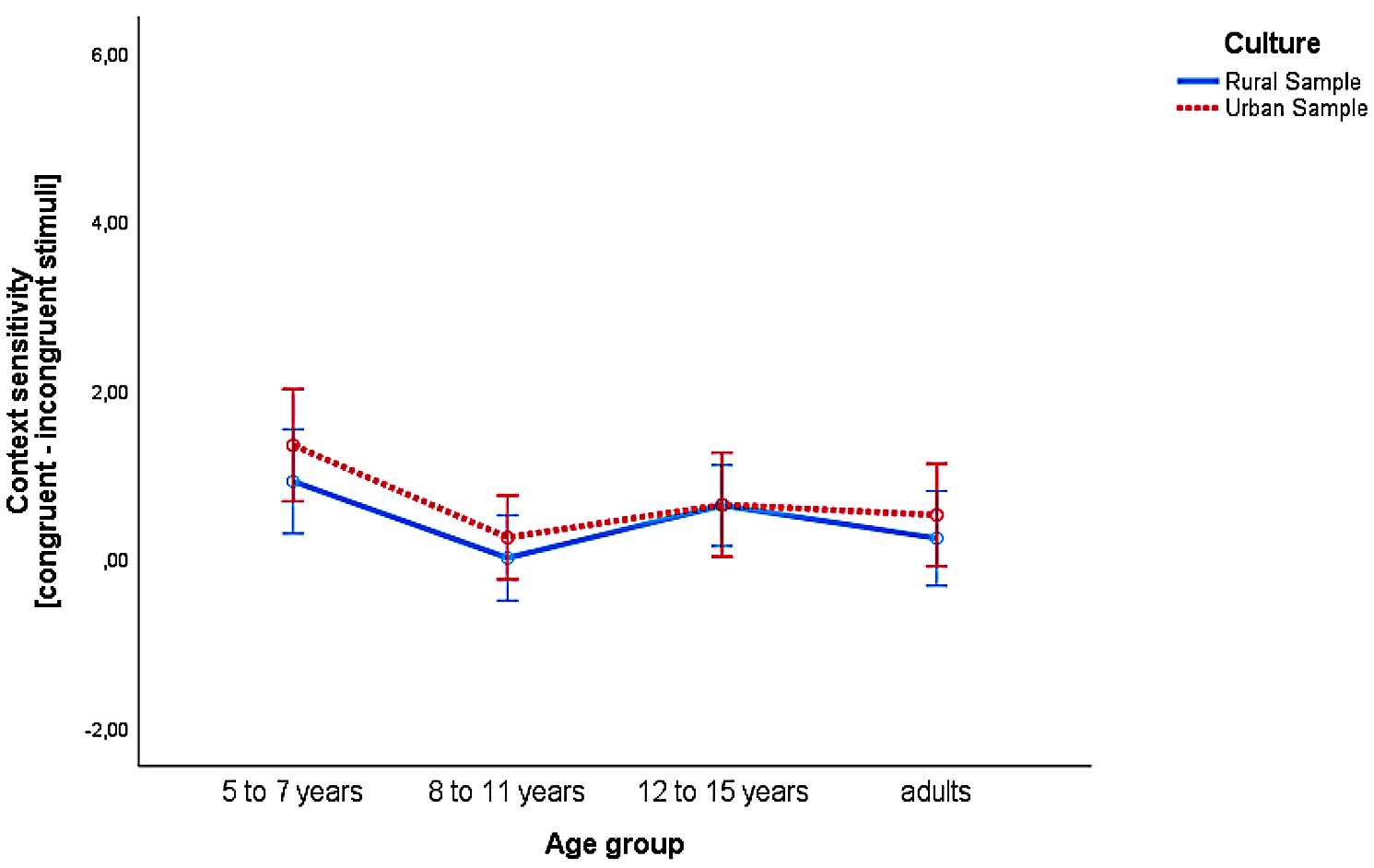


***Supplementary Figure 1.*** Context-sensitivity scores by culture and age group for the sad stimuli set in the judgment of facial emotions task. Higher values indicate higher context sensitivity. Whiskers depict one standard error of the mean.
